# Atypical Hemolytic Uremic Syndrome (aHUS) and Adenosine Deaminase (ADA)-Deficient Severe Combined Immunodeficiency (SCID)—Two Diseases That Exacerbate Each Other: Case Report

**DOI:** 10.3390/ijms22179479

**Published:** 2021-08-31

**Authors:** Anna Bogdał, Andrzej Badeński, Małgorzata Pac, Anna Wójcicka, Marta Badeńska, Agnieszka Didyk, Elżbieta Trembecka-Dubel, Nel Dąbrowska-Leonik, Małgorzata Walaszczyk, Natalia Matysiak, Aurelia Morawiec-Knysak, Tomasz Szczepański, Maria Szczepańska

**Affiliations:** 1 District Hospital in Zawiercie, ul. Miodowa 14, 42-400 Zawiercie, Poland; ania.bog94@wp.pl; 2 Department of Pediatrics, Faculty of Medical Sciences in Zabrze, Medical University of Silesia in Katowice, ul. 3 Maja 13/15, 41-800 Zabrze, Poland; badenski.andrzej@gmail.com (A.B.); marta.badenska2@gmail.com (M.B.); 3 Department of Immunology, The Children’s Memorial Health Institute, 04-730 Warsaw, Poland; m.pac@ipczd.pl (M.P.); nel.dabrowska@wp.pl (N.D.-L.); 4 Warsaw Genomics, 01-682 Warsaw, Poland; anna.wojcicka@warsawgenomics.pl; 5 Department of Pediatric Nephrology with Dialysis Division for Children, Public Clinical Hospital No. 1 in Zabrze, 41-800 Zabrze, Poland; adidyk90@gmail.com (A.D.); etdubel@interia.pl (E.T.-D.); aurelia.knysak@gmail.com (A.M.-K.); 6 Department of Anaesthesiology and Intensive Therapy, Faculty of Medical Sciences in Zabrze, Medical University of Silesia in Katowice, ul. 3 Maja 13/15, 41-800 Zabrze, Poland; gosiekprom@wp.pl; 7 Department of Histology and Cell Pathology, Faculty of Medical Sciences in Zabrze, Medical University of Silesia in Katowice, ul. 3 Maja 13/15, 41-800 Zabrze, Poland; nmatysiak@sum.edu.pl; 8 Department of Pediatric Hematology and Oncology, Faculty of Medical Sciences in Zabrze, Medical University of Silesia in Katowice ul. 3 Maja 13/15, 41-800 Zabrze, Poland; tszczepanski@sum.edu.pl

**Keywords:** atypical hemolytic uremic syndrome, thrombotic microangiopathy, severe combined immunodeficiency, adenosine deaminase deficiency, children

## Abstract

Hemolytic uremic syndrome (HUS) is defined by the triad of microangiopathic hemolytic anemia, thrombocytopenia, and acute kidney injury (AKI). Atypical HUS (aHUS), distinguished by its etiology, is caused by uncontrolled overactivation of the alternative complement pathway. The correct diagnosis of aHUS is complex and involves various gene mutations. Severe combined immunodeficiency (SCID), characterized by severe T-cell lymphocytopenia and a lack of antigen-specific T-cell and B-cell immune responses, is of seldom occurrence. In 10–15% of pediatric patients, SCID is caused by adenosine deaminase (ADA) deficiency. The authors describe the case of a boy who suffered from both aHUS and ADA-deficient SCID. At the age of 9 months, the patient presented acute kidney injury with anuria and coagulopathy. The diagnosis of aHUS was established on the basis of alternative complement pathway deregulation and disease-associated gene mutations. Further examination revealed immune system failure and, at the age of 13 months, the ADA deficiency was confirmed by genetic tests and the boy was diagnosed with ADA-SCID. ADA SCID has recently been described as a possible triggering factor of aHUS development and progression. However, more research is required in this field. Nevertheless, it is crucial in clinical practice to be aware of these two co-existing life-threatening diseases.

## 1. Introduction

Atypical hemolytic uremic syndrome (aHUS), a rare and severe disease, belongs to a family of thrombotic microangiopathies (TMAs). It is caused by uncontrolled, incessant activation of the alternative complement pathway, on account of defects in its regulation. Usually, aHUS is induced by the presence of genetic variants that diminish the function of complement regulating factors (e.g., factor H and I (CFH, CFI), and membrane cofactor protein (MCP = CD46)), as well as through activating mutations of C3 or complement factor B (CFB) or other related coagulation factors. Autoantibodies against CFH can provoke a clinical phenotype similar to aHUS, especially with the presence of CFH-related factor mutations. All children with HUS should also be tested for possible cobalamin C deficiency [1]. aHUS is characterized by the classic triad of symptoms: microangiopathic hemolytic anemia (MAHA), thrombocytopenia, and acute kidney injury. Renal manifestations of the disease are described as fibrin and platelet thrombi in capillaries and arterioles, endothelial cell swelling and detachment from the glomerular basement membrane, and the appearance of so-called double contours on the glomerular basement membrane [2].

Adenosine deaminase (ADA) deficiency, owing to *ADA* gene mutation, results in the purine metabolism disorder. The accumulation of toxic metabolites, such as deoxyadenosine triphosphate and deoxyadenosine, is the main pathogenic mechanism. Such a disruption affects various tissues, as ADA is ubiquitously expressed throughout the body; however, it profoundly impacts the immune system [3]. Consequently, the development and functioning of lymphocytes are impaired, finally causing severe combined immunodeficiency (SCID) [4]. A large population-based newborn screening ascertainment revealed that ADA deficiency occurs in 1 per 500,000 cases and accounts for 10–20% of human SCID. SCID is characterized by severe T-cell lymphocytopenia and a lack of antigen-specific T-cell and B-cell immune responses. This condition is uniformly fatal in the first two years of life unless immune reconstitution can be accomplished with allogeneic hematopoietic stem cell transplantation or gene therapy. ADA-SCID, being a non-lymphocyte-specific cause of immunodeficiency, is unique among the different genetic causes of SCID [5].

### Aim

This manuscript presents the case of a boy who suffered from both aHUS and ADA SCID—two diseases that exacerbated each other.

## 2. Clinical Report

The boy was born to healthy Caucasian parents after the second pregnancy and the second delivery, at the 38th week of gestational age. The delivery was uncomplicated with a birth weight of 3150 g, body length of 54 cm, and an APGAR score of 9. Since infancy, the patient was presenting recurrent diarrhea, resulting in impaired weight gain—his weight remained at the third percentile for age and sex. Consequently, a retardation of psychomotor development was assumed.

**Figure 1:**
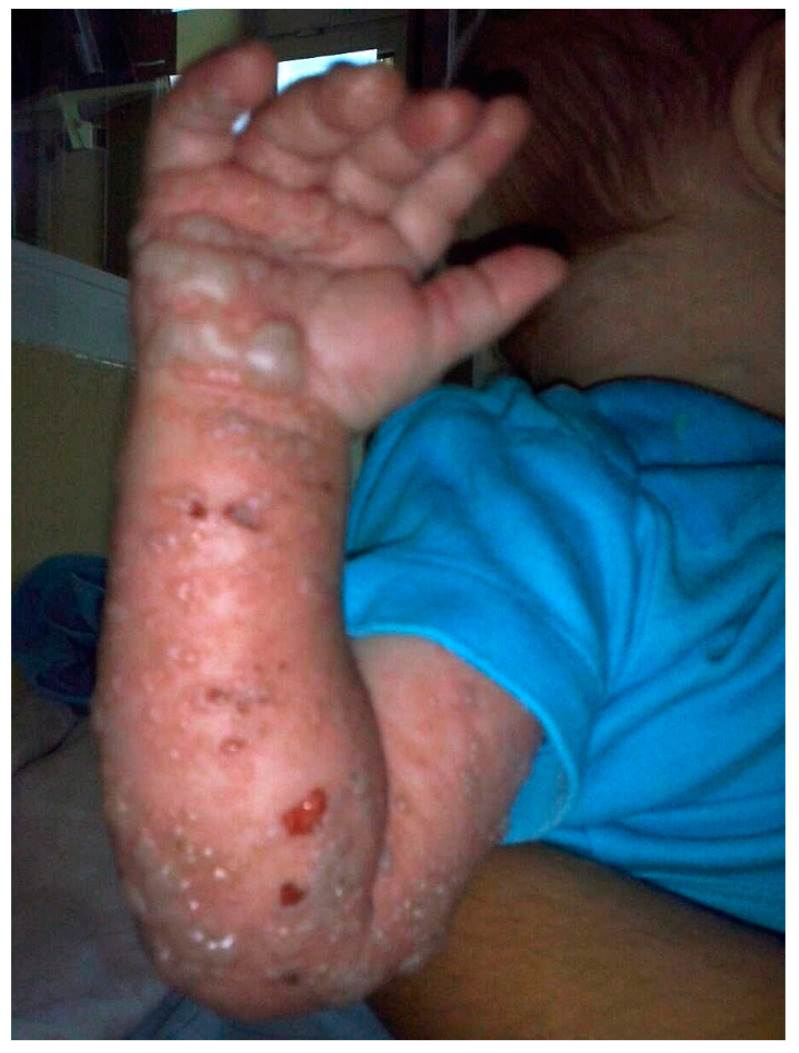
Severe course of Varicella Zoster in the described case.

At the age of three months, he developed varicella and, six months later, herpes zoster (Figure 1). The patient presented with anuric acute kidney injury, generalized edema, and respiratory failure, requiring immediate medical intervention. On admission to the intensive care unit, laboratory tests revealed various significant abnormalities (Table 1). An elevated reticulocytes count, hyperbilirubinemia, elevated serum creatinine, and increased lactate dehydrogenase (LDH) activity, together with proteinuria and hematuria, raised the suspicion of the diagnosis of HUS. Moreover, schistocytes were present in peripheral blood smear, accounting for 6% of RBC, and elevated aspartate transaminase (AST), alanine transaminase (ALT), gamma-glutamyl transferase (GGT), and c-reactive protein (CRP) values were noted. Owing to the severe clinical condition, the boy required peritoneal dialysis (PD) performed continuously, starting from hourly exchanges of dialysis fluid. In addition, owing to aHUS suspicion and coagulopathy (thrombocytopenia, prolonged activated partial thromboplastin time (aPTT) and prothrombin time (PT) along with reduced fibrinogen activity), blood product transfusions were performed. Shiga toxin-producing *Escherichia coli* infection was excluded by fecal culture. Furthermore, assessment of complement components indicated slightly decreased C3 levels together with C4 levels within the normal range. ADAMTS13 (disintegrin and metalloproteinase with a thrombospondin type 1 motif member 13) activity was 60%.

PD continued for 29 days (969 h), and blood plasma transfusions and pharmacological treatment did not provide a significant improvement in the patient’s condition. Consequently, it was decided to conduct eight cycles of plasmapheresis, eventually resulting in a good clinical response (that time eculizumab treatment was unavailable) (Table 1). Three sessions of plasmapheresis were performed daily, and another five on alternating days. The amount of exchanged plasma was on average 600 mL/session (80 mL/kg/body weight). Additionally, the boy received combined hypotensive therapy, including amlodipine, bisoprolol, and ramipril. For the next month, the patient remained under outpatient nephrological care.

**Figure 2:**
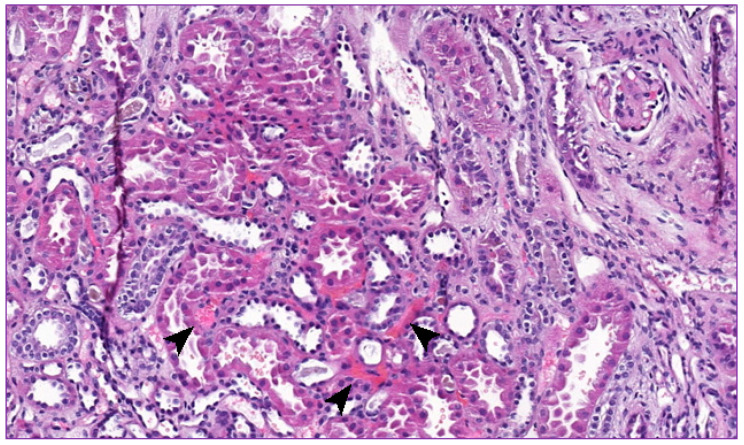
Small fibrin thrombi are visible in the interstitium (arrowheads).

**Figure 3:**
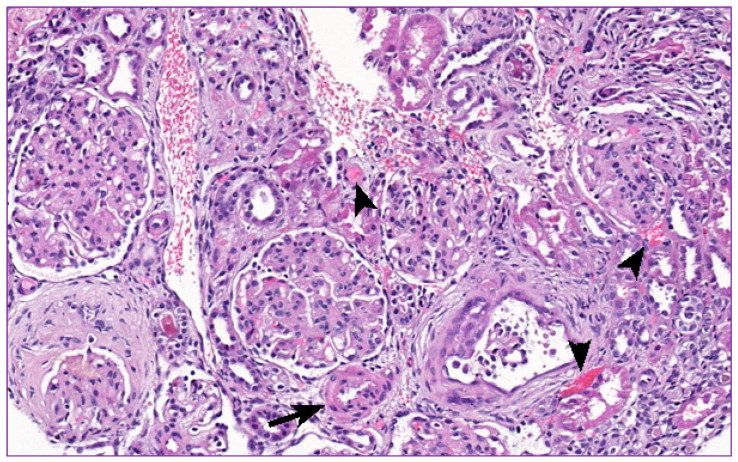
Increased cellularity of the glomeruli, accompanied by extracellular matrix expansion. Small thrombi located in the interstitium (arrowheads). Small arteriole with a thick wall (arrow).

**Figure 4:**
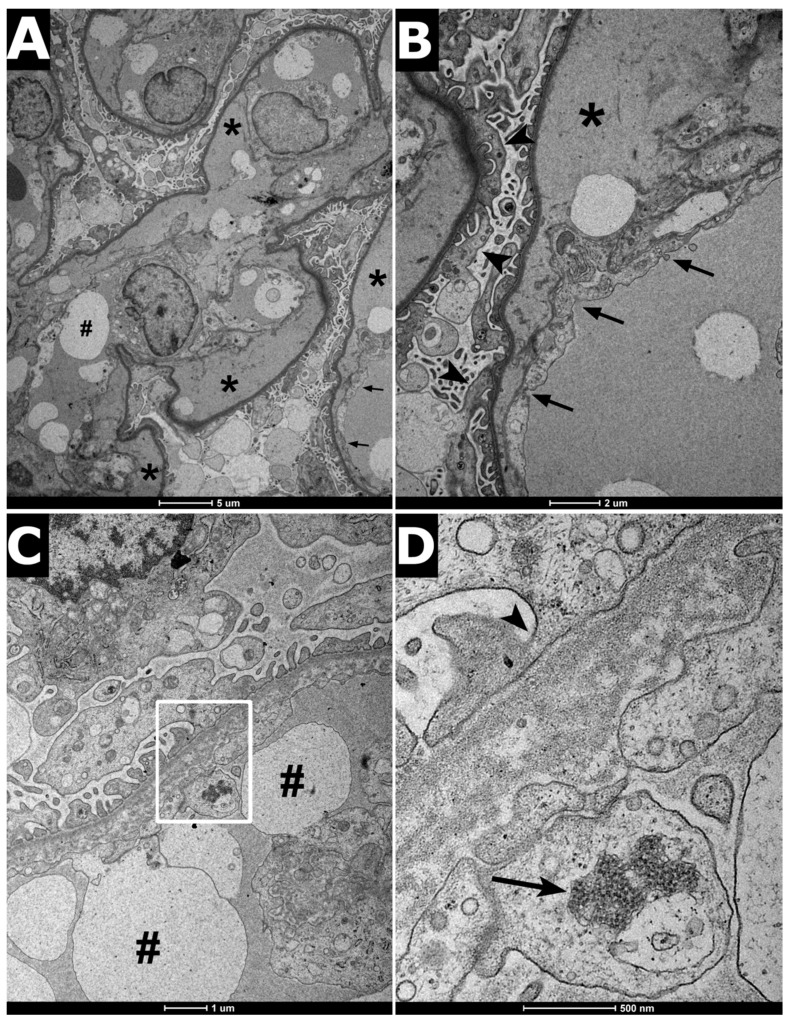
Electron microscopy findings in HUS. (**A**) Glomerular capillary separation of endothelial cells from the underlying glomerular capillary basement membrane (asterisks), markedly widened sub-endothelial space with fluffy-like material. Swelling of the capillary endothelial cells, electron lucent blebs are visible in the capilary lumen (#). Scale bar: 5 μm. (**B**) Higher magnification of a capillary wall; the arrows indicate the loss of endothelial fenestration, foot processes flattening, and microvilli on podocyte surface are also visible (arrowheads). Scale bar: 2 μm (**C**) Fragment of glomerular capillary wall with a widened sub-endothelial space and rich fluffy-like material under the endothelial cells. Capillary loop exhibits mild endothelial cell swelling with the absence of normal fenestra (#) Scale bar: 1 μm. (**D**) White box, magnification of (**C**) Endothelial cell contains a tubuloreticular inclusions (arrows). Moreover, flattening of podocyte foot processes also occurs (arrowhead). The presence of tubuloreticular inclusions (TRIs) in glomerular endothelial cells associated with viral infections (so-called interferon footprints) Scale bar: 500 nm.

At the age of 13 months, the boy was admitted to the hospital because of high body temperature and severe dehydration accompanied by elevated AST, GGT, and CRP levels (Table 1). Further examination revealed an upper urinary tract infection (pharyngotonsillitis), followed by increased generalized edema, low urine output, and hypoalbuminemia in subsequent days. Consequently, amoxicillin with clavulanic acid, furosemide, and 20% albumin solution was administered. Unfortunately, the treatment was unsuccessful and, eventually, left-sided partial seizures, decorticate posturing, and opisthotonus occurred. Head computed tomography (CT) and cerebrospinal fluid analysis were performed, yet no abnormalities were found. Additionally, in the echocardiogram, a sinus venosus, an atrial septal defect, and a slight tricuspid valve regurgitation were present. In the following days, symptoms of pulmonary edema arose and the boy required PD and mechanical ventilation for 6 days. Further tests revealed a serum creatinine level of 37.7 µmol/L and urea concentration of 8.7 mmol/L. Platelet count was maintained in the range of 315–444 10^3^/μL. A successful treatment was followed merely by several days of the patient’s good condition. Pneumonia accompanied by severe anemia was disclosed within days. During blood transfusion, a sudden cardiac arrest occurred and the boy required intensive care once more. As soon as his condition improved, a renal biopsy was conducted, revealing mesangial cell proliferation accompanied by hyaline degeneration, multiple hyaline casts in the glomerular lumen, and thickening within each layer of the arterial wall. Such a description strongly corresponded with membranoproliferative glomerulonephritis (MPGN) and vasculopathy (Figure 2 and Figure 3). A part of the glomerular capillary wall with a widened sub-endothelial space and rich fluffy-like material beneath the endothelial cells was described in the performed electron microscopy examination (Figure 4). Bone marrow examination, conducted to broaden the diagnostic process, revealed 23% of lymphoblast-like cells, while precursor B cells in various development stages represented 13% of cells in immunophenotyping. No mature B cells were detected, neither in the bone marrow nor in peripheral blood.

Within a few days after renal biopsy, an appearance of blood in the urine was noticed and abdominal ultrasound revealed an arteriovenous fistula of the right kidney; therefore, the boy underwent successful embolization of the damaged artery.

Suspecting the presence of aHUS and considering its genetic basis, genetic testing was performed by Warsaw Genomics. *ADAMTS13; C3; C5; CD46; CFB; CFH; CFHR1; CFHR2; CFHR3; CFHR4; CFHR5; CFI; DGKE; MMACHC; PLG; THBD* genes were analyzed. Analysis of large deletions and rearrangements did not disclose any mutations in *CFHR1-5 gene*, as well as *DGKE; MMACHC; PLG; THBD, C3, C5, CFI genes*. Point mutation, included in the high-risk haplotype for aHUS and associated with a poor prognosis and greater risk of transplant rejection, was detected in *CFH* gene (TGTGT) [6]. Additionally three high-risk haplotypes were found in *CD46* gene (rs2796267, rs2796268, and rs859705) [7,8]. Both copies of *ADAMTS13 gene* were described with *the rs2301612* variant, previously detected in patients with aneurysms [9]. Furthermore, another variant, quite rare in the general population (MAF = 0.007 in the GnomAD database), was discovered in a single *CFB gene* copy (rs13194698; NM_001710.5:c.299-111C>T), yet its clinical manifestation is still unknown (Table 2 genes are listed in alphabetical order).

The patient demonstrated persistent lymphopenia with absolute lymphocyte numbers ranging from 0.17 to 0.8 × 10^3^/μL (N: 1.0–4.4 × 10^3^/μL). The immunophenotyping of peripheral blood was performed with multicolor flow cytometry and the lymphocyte subsets were identified according to the EuroFlow standards [10]. This investigation showed decreased numbers of B-cells, T-cells, and NK-cells, which was suggestive of T^-^B^-^NK^-^ SCID (Table 3) and prompted further investigation at the specialized institute. CD45+ cells unveiled RO surface antigen at 95.5%. The levels of all main subclasses of immunoglobulins were significantly below the normal range.

Concomitantly, genetic tests unraveled homozygous pathogenic mutation in *ADA gene* (c.302G > A, (p.Arg101Gln)). The clinical outcome and test results indicated adenosine deaminase (ADA)-deficient severe combined immune deficiency (SCID). Additionally, other immunodeficiency connected genes were analyzed and are presented in the Supplementary Material (Table S1). No further abnormalities were found.

At the age of 16 months, the boy was admitted to the district hospital because of a severe respiratory tract infection. Unfortunately, a progression of the infection with the severity of the underlying pathology led to multiorgan failure and the patient deceased.

## 3. Discussion

### 3.1. Atypical Hemolytic Uremic Syndrome

Microangiopathic hemolysis (MAHA) was the initial diagnosis in the presented case. According to the current pathophysiological classification of TMAs, three main types are distinguished: primary (congenital and acquired), secondary, and infection induced. To exclude thrombotic thrombocytopenic purpura (TTP), ADAMTS13 activity was performed, which showed the level above 10%. HUS induced by Shiga toxin-producing bacteria (STPB, predominantly Shiga toxin-producing Escherichia coli (STEC); STEC HUS), classified as infection-induced TMA, is the most common type among young patients [2,11]. Thus, the main step in differential diagnosis of aHUS in children is the exclusion of STEC HUS. Therefore, fecal culture examination was performed in the described case, eliminating infection caused by verotoxin-producing bacterial strains. Furthermore, the combination of low C3 and normal C4 supported the diagnosis of aHUS. Kidney biopsy was also helpful in the diagnostic process. Description of membranoproliferative glomerulonephritis (MPGN) with the presence of thrombotic microangiopathy and fibrin deposits also supported aHUS-related pathology.

Although there has been substantial progress in this field of study, for about 40% of patients, the etiology of aHUS is still unknown [12]. Currently, aHUS diagnosis is still difficult to establish clearly, as no direct diagnostic test exists and available biomarkers are not completely reliable [2,11]. Moreover, the detection of complement gene variants is an obligatory element of the diagnostic process. Among aHUS-related gene disorders, *CFB, CFH,* and *MCP gene* mutations were found in the presented patient. Complement factor H haplotype TGTGT is a modest risk factor OR1.89 according to de Cordoba et al. [6]. Detected MCP gene mutations in our patient in accordance with previous findings of Fremeaux-Bacchi show that genetic variability in MCP is also associated with atypical HUS in that case [7].

In a differential diagnosis, we excluded the mutations in diacylglycerol kinase epsilon (DGKE) gene, whose presence could be responsible for the renal pathology of thrombotic microangiopathy or MPGN [13,14] and cobalamin C disease, which may also present as HUS with renal TMA during infancy [15]. Additionally, copies of ADAMTS13 gene were described with the rs2301612 variant defined before in patients with aneurysms, which is a benign variant that does not decrease ADAMTS13 activity [9,16]. In CT imaging the aneurysms were excluded in the presented patient. Such a variant of *ADAMTS13 gene* is frequently observed among patients with aHUS in our department.

Performing genetic tests should not delay the treatment of aHUS. Therefore, the described patient was given a suitable therapy in compliance with standards of care for the first presentation of aHUS, receiving plasma exchange therapy (PLEX), as eculizumab treatment was not yet available in Poland at that time [17].

In the described case, the incident of low urine output causing severe overhydration was defined as an exacerbation of chronic kidney disease during septic infection. Occurring reversible neurological complications with negative head CT scan, pulmonary edema, worsening anemia, and cardiac arrest were not related to aHUS.

### 3.2. Adenosine Deaminase (ADA)-Deficient Severe Combined Immune Deficiency (SCID)

In the presented case, genetic tests revealed homozygous pathogenic mutations in ADA gene that eventually led to purine metabolism disorder.

ADA deficiency is said to be the first acknowledged underlying pathology of SCID [4]. It is directly associated with a systemic purine metabolic disorder that is described to impact various organ systems, such as pulmonary, hematologic, gastrointestinal, neurologic, or skeletal [5]. Among the symptoms, hepatotoxicity (including hepatitis, hyperbilirubinemia, or even rapid hepatic failure) is frequently observed; likewise, it was described in the presented case. The differential diagnosis of increased activity of hepatic enzymes should be performed towards infectious, autoimmune, metabolic, and toxic causes, especially before establishing the diagnosis of ADA-SCID [18,19]. In the case of our patient, hepatotropic infections as well as autoimmune and common metabolic diseases were excluded. Pulmonary alveolar proteinosis of ADA-SCID should also be taken into consideration during differential diagnosis of pulmonary edema, especially when the *Pneumocystis jiroveci* infection has been excluded.

The lymphocyte functioning disorder due to ADA deficiency has already been deeply explored; however, other systemic manifestation mechanisms have not yet been fully understood. In the literature, a number of purine metabolism disorders were proved to indicate immunodeficiency [20]. ADA is a major enzyme in irreversible deamination of purine nucleosides, such as adenosine (Ado) into inosine and 2′-deoxyadenosine (d-Ado) into 2′-deoxyinosine (d-Ino) [21,22]. In the absence of this reaction in purine metabolism, d-Ado and Ado accumulate in fluids and cellular components of affected individuals, causing immune cell impairment, mainly affecting the development and function of T, B, and NK cells and, furthermore, systemic pathology [23,24]. The patient presented with profound lymphopenia from the beginning. Moreover, in conformity with the literature, he revealed an elevated percentage of memory CD45RO+ lymphocytes. It is worth mentioning that PLEX and repeated plasma transfusions, applied for aHUS treatment, might have provided the patient with passive humoral immunity.

Apart from immune system defect, Ado accumulation might be responsible for CNS complications. In our patient, a negative head CT scan documented only functional injury at the initial SCID presentation.

In genetic evaluation, we have presented the mutation in *ADA* gene and negative results in the analysis of genes connected with other congenital immunodeficiencies. Such a detailed evaluation has not been done previously.

Nowadays, early ADA restoration, by either enzyme replacement therapy, allogeneic hematopoietic stem cell transplantation (HSCT), or gene therapy, is available and prevents patients from severe immunodeficiency [3].

### 3.3. ADA-SCID and aHUS Interaction

According to the literature, which is limited to case reports, the mechanism of ADA-SCID influencing aHUS development might be connected with infection, deregulation of complement function, autoimmunity, or impaired metabolism; however, no certain connections were established [4,25]. However, these reports cannot be taken as evidence that the reported cases did have thrombotic microangiopathy due to defective regulation of the alternative complement pathway, because genetic variants were not demonstrated. We have tried to find the genetic variants confirming defective regulation of the alternative complement pathway in ADA-SCID child and TMA for the first time.

Constriction of afferent renal arterioles and subsequent glomerular filtration reduction are the consequences of signaling through the adenosine A1 receptor [26,27]. Therefore, an elevated extracellular adenosine level within renal tissue in ADA-deficient patients is suspected to gradually induce nephrotic proteinuria, as ADA-SCID patients’ autopsy reports disclosed mesangial sclerosis in the majority of cases and global glomerular sclerosis in up to 10% of glomeruli on death [28]. Such findings may impact the course of aHUS, accelerating the progress of renal failure and diminishing the clinical response to treatment. The findings of this case, together with the cases described in the cited references [4,25], suggest that ADA-SCID apart from aHUS may present with nephropathy during infancy, also owing to adenosine-mediated arterial and glomerular injury with concurrent hepatic injuries.

The obligatory combination of factors for aHUS to develop is believed to include infections, genetic disorders, and environmental susceptibility [29,30]. Hence, herpes zoster infection, presented in this case, was considered the triggering factor of aHUS development. Disseminated varicella caused by wild-type virus or following varicella vaccine in SCID patients, including ADA-SCID, have already been reported [31]. In our case, the mother’s medical history concerning either VZ infection or vaccination remains unknown, which precludes the exact explanation of such an early infection. It is believed that ADA-SCID patients, owing to immune system abruption, are more likely to develop aHUS as a result of disease caused by a wider range of pathogens, or even an opportunistic infection.

As mentioned above, purine metabolism disorders caused by ADA deficiency might influence various tissues by currently unknown mechanisms. It is assumed that biologically active purine metabolites, accumulating in the organism, may trigger metabolic changes, which consequently might contribute to the predisposition to aHUS development, as was also mentioned in the study of Nikolajeva et al. [4].

## 4. Conclusions

In clinical practice, ADA-SCID patients should be carefully examined in terms of aHUS development possibilities. Furthermore, early treatment in this group of patients is essential, as both conditions, described as severe and life-threatening, are considered to exacerbate each other. However, currently, various therapies are available and should be induced as soon as possible.

## Figures and Tables

**Table 1:** A,B. Laboratory test results. A—hospitalization at the age of 9 months, B—hospitalization at the age of 13 months.

Laboratory Tests	A	B	Reference Ranges
**Complete Blood Count** On admission On discharge			
Hematocrit [%]	32.5, 30.7	32.25	32–42
Red blood cell count [10^6^/uL]	4.98, 3.52	4.05	3.8–5.4
Hemoglobin [g/dL]	11.3, 10.2	11.98	10.5–14
White cell count [10^3^/uL]	3.79, 5.26	4.53	6–14
Granulocytes [10^3^/uL]	1.35, 1.76	2.7	1.6–6
Lymphocytes [10^3^/uL]	0.46, 0.93	0.4	1.0–3.3
Monocytes [10^3^/uL]	1.56, 0.42	1.2	0.15–0.6
Mean corpuscular volume [fL]	65.3, 87.22	87.13	72–88
Mean corpuscular hemoglobin [pg]	22.7, 28.92	29.62	24–30
Mean corpuscular hemoglobin concentration [g/dL]	34.8, 33.16	34	32–36
Platelet count [10^3^/uL]	50, 361	379	150–450
Reticulocyte count [10^9^/L]	87.6	70.2	26–85
**Serum laboratory tests**			
Albumins [g/L]	29.84, 38.44	29.65	30–50
Aspartate aminotransferase [U/L]	660.6, 104.3	51.2	0.40
Alanin aminotransferase [U/L]	178.1, 49.2	33.2	0–40
Bilirubin (total) [umol/L]	22.8, 3.9	0.5	3.4–22
Complement component 3 [g/L]	0.84, 0.86	-	0.9–1.8
Complement component 4 [g/L]	0.1, 0.28	-	0.1–0.4
C-reactive protein [mg/L]	23.56, 2.63	37.95	0–5
Gamma glutamyltranspeptidase [U/L]	198, 127	360	5–65
Total protein [g/L]	41.1, 62.8	62.2	60–80
Creatinine [umol/L]	149, 61	55	21–53
Uric acid [umol/L]	-, 226	243	202.3–416.5
Urea [mmol/L]	23.1, 4.9	20.7	3.2–7.1
Lactate dehydrogenase [U/L]	3757, 591	-	180–435
Sodium [mmol/L]	121.1, 136	135	135–148
Potassium [mmol/L]	5.43, 5.84	3.74	3.5–5.0
Magnesium [mmol/L]	0.78, 0.87	1.05	0.7–1.1
Phosphate [mmol/L]	1.15, 1.74	1.02	0.81–1.45
Ionised calcium [mmol/L]	0.86, 1.19	1.11	1.1–1.35
Proteinuria [g/L]	26.0, 1.35	0.75	0–0.15
Hematuria [RBC/HPF]	Massive, 5–10	1–3	0–3
**Coagulation profile**			
Activated partial thromboplastin time [s]	40.2, 38.1	27.8	28–40
Prothrombin time [s]	16.3, 13.1	12.5	11–16
International normalized ratio [INR]	1.31, 0.99	0.95	0.9–1.3
Fibrinogen [mg/dL]	178, 347	252	200–400
D-dimer [mg/mL]	3.1, 1.76	1.05	0–0.5
Antithrombin III [%]	90, 110	81	80–120

**Table 2:** Genetic testing.

Gene	Description	Inheritance	Additional Information
*ADAMTS13*	allele G rs2301612	mutations in both copies of gene	maternal and paternal
*CD46*	allele A rs2796267	mutations in both copies of gene	maternal and paternal
*CD46*	allele A rs2796268	mutations in both copies of gene	maternal and paternal
*CD46*	allele G rs859705	mutations in both copies of gene	maternal and paternal
*CFB*	allele T rs13194698	mutations in single copy of gene	maternal
*CFH*	allele T rs3753394	mutations in single copy of gene	paternal
*CFH*	allele G rs800292	mutations in both copies of gene	paternal
*CFH*	allele T rs1061170	mutations in single copy of gene	paternal
*CFH*	allele G rs3753396	mutations in single copy of gene	paternal
*CFH*	allele T rs1065489	mutations in single copy of gene	paternal
*CFH*	allele A rs2274700	mutations in single copy of gene	maternal and paternal

**Table 3:** Immunological tests.

Lymphocyte Immunophenotyping			Immunoglobulins	[g/L]
Parameter	Absolute Number [10^3^/uL]	Percentage of Lymphocytes		
T-cells	0.11 [N: 1.6–6.7]	79%	IgG	3.09 [N: 4.53–9.16]
B-cells	0.0056 [N: 0.6–2.7]	0.04%	IgA	0.11 [N: 0.19–1.46]
NK-cells	0.0287 [N; 0.2–1.2]	20.5%	IgM	0.1 [N: 0.2–1]
CD4 T-cells	0.00002 [N: 1.4–5.1]	1.2% of T-cells		
CD8 T-cells	0.10 [N: 0.6–2.2]	95.5% of T-cells		

The ranges of the presented parameters were adjusted to the child’s age.

## Supplementary Materials

Supplementary materials can be found at https://www.mdpi.com/article/10.3390/ijms22179479/s1.
