# Supplementary material for: Atypical Hemolytic Uremic Syndrome (aHUS) and Adenosine Deaminase (ADA)-Deficient Severe Combined Immunodeficiency (SCID)—Two Diseases That Exacerbate Each Other: Case Report
Source: Int J Mol Sci. 2021 Aug 31;22(17):9479. doi: 10.3390/ijms22179479 (PMC8430959; doi:10.3390/ijms22179479)
Supplement: Supplementary file 1 [file ijms-22-09479-s001.zip › ijms-1320393-supplementary.pdf]

Other analyzed immunodeficiency connected genes:

ADAR; C1QC; C1R; C1S; C2; C3; C3AR1; C4A; C4B; C4BPA; C4BPB; C5; C5AR1; C5AR2; C6; C7; C8A; C8B; C8G; C9; CARD11; CARD14; CASP10; CASP8; CCDC103; CCDC114; CCDC39; CCDC40; CCDC65; CCNO; CD19; CD247; CD27; CD3D; CD3E; CD3G; CD40; CD40LG; CD46; CD55; CD59; CD8A; CD93; CECR1; CFD; CFP; CIITA; CLU; COLEC11; CORO1A; CR1; CR2; CRP; CSF2RA; CTC1; CTLA4; CTSC; CYBA; CYBB; DCLRE1C; DDX58; DGKE; DKC1; DNAAF1; DNAAF2; DNAAF3; DNAAF5; DNAH11; DNAH5; DNAI1; DNAI2; DNAL1; DNMT3B; DOCK2; DOCK8; DRC1; DYX1C1; ELANE; FAS; FCN1; FCN2; FCN3; FERMT3; FOXP3; G6PC3; G6PD; GATA2; HAX1; HYDIN; IFIH1; IFNGR1; IFNGR2; IGHM; IGLL1; IKBKG; IL10RA; IL17RA; IL10RB; IL12RB1; IL1RN; IL2RA; IL2RG; IL36RN; IL7; IL7R; ISG15; ITGB2; ITK; JAGN1; JAK3; LCK; LIG4; LPIN2; LRBA; LRRC6; LYST; MAGT1; MALT1; MASP1; MASP2; MAT2A; MEFV; MRE11; MVK; NBN; NCF1; NCF2; NCF4; NFKB1; NFKB2; NFKBIA; NHEJ1; NHP2; NLRP12; NLRP3; NME8; NOD2; NOP10; NRAS; OFD1; ORAI1; PIGA; PIK3CD; PIK3R1; PLCG2; PMS2; PNP; PRF1; PRKDC; PSMB8; PSTPIP1; PTPRC; PTX3; RAB27A; RAG1; RAG2; RFX5; RFXANK; RFXAP; RHOH; RMRP; RNASEH2A; RNASEH2B; RNASEH2C; RPGR; RSPH1; RSPH4A; RSPH9; RTEL1; SAMHD1; SBDS; SERPING1; SH2D1A; SLC37A4; SMARCAL1; SP110; SPAG1; SPINK5; STAT1; STAT2; STAT3; STAT4; STAT5B; STIM1; STK4; STXBP2; TAP1; TAP2; TAPBP; TBC1D27; TBX1; TCIRG1; TERC; TERT; THBD; TINF2; TMEM173; TNFRSF13B; TNFRSF1A; TNFRSF4; TRAC; TREX1; TYK2; UNC119; USB1; VSIG4; VTN; WAS; WRAP53; XIAP; ZAP70; ZMYND10.
